# Sex, Gender, and the Search for Analytic Truth

**DOI:** 10.1002/jmri.70399

**Published:** 2026-06-13

**Authors:** Jitka Starekova, Mark E. Schweitzer

**Affiliations:** ^1^ Department of Radiology University of Wisconsin‐Madison Madison Wisconsin USA; ^2^ Health Affairs Office Wayne State University Detroit Michigan USA

**Keywords:** gender, neuroimaging, research methodology, sex

Over the past decade, the distinction between “sex” and “gender” has shifted from an implicit assumption to an explicit requirement in both the common vernacular as well as in medical care and especially research. Historically, these terms were often used interchangeably, with “sex” serving as a default descriptor without clarification of whether it referred to chromosomal, hormonal, anatomical, or self‐reported characteristics. Increasing emphasis on both inclusivity and in terms of medical journals, scientific rigor has prompted a need and expectation of a clearer differentiation: *sex* should refer to biological attributes such as chromosomes, reproductive anatomy, and hormonal milieu, whereas *gender* describes identity and sociocultural context.

This shift is now reflected in major editorial and policy frameworks. Guidelines such as the American Medical Association (AMA) Manual of Style and Gender Equity in Research (SAGER) recommendations advocate for precise terminology, clear definitions, and transparent reporting [1, 2]. Without such distinctions, research risks conflating biological and sociocultural effects, potentially biasing data interpretation.

While it is somewhat clear that when imaging the prostate or breast, usually, sex should be reported, neuroimaging research is more complex. The brain is both biologically determined and highly plastic, with function shaped by genetic factors and hormonal exposure, as well as environment, stress, and social experience. As a result, imaging‐derived measures, whether structural, functional, or connectivity‐based, cannot be readily attributed to biological sex or gender alone.

Biological sex remains a critical variable in neurological and psychiatric research. Epidemiological differences are well recognized; for instance, multiple sclerosis is more prevalent in females [3], whereas Parkinson's disease is more common in males [4]. These patterns are often interpreted as evidence of underlying biological divergence, potentially mediated by immune modulation, mitochondrial function, or hormonal influences. Neuroimaging studies have similarly reported sex‐associated differences in brain morphology and function, including in regions such as the amygdala and hippocampus [5].

While the epidemiological disparities in diseases like Parkinson's highlight undeniable sex‐specific biological vulnerabilities, the structural brain findings require more careful interpretation. Increasing evidence suggests that the human brain is not strictly dimorphic but rather a mosaic of features, with substantial interindividual overlap. Moreover, sex rarely acts in isolation. Genetic predisposition, environmental exposures, and healthcare access interact in complex ways, and in some cases may exert a stronger influence on disease natural history than sex alone [6]. Overreliance on binary biological categories (male/female) therefore risks oversimplifying these interactions.

Hormonal dynamics add to this complexity. Exposure to sex hormones during critical developmental windows contributes to neuronal differentiation, synaptic pruning, and network formation [7]. Yet these environments are dynamic rather than fixed. Endogenous changes, such as those occurring during puberty, pregnancy, or menopause, and exogenous factors, including therapies, can alter brain structure and function across the lifespan. Neuroimaging studies in transgender individuals, for example, show that gender‐affirming hormone therapy is associated with measurable changes in cortical thickness and functional connectivity [8]. These findings underscore the brain's ongoing plasticity and challenge static categorizations based solely on sex assigned at birth.

At the same time, gender‐related experiences exert their own influence on the brain. Social roles, stress exposure, individual and societal behavior, and cultural expectations can (re)shape CNS organization through neuroplasticity. Because of this, neuroimaging signals often capture both the effects of biology and lived experience, which are not easily separated by a specific descriptor. Importantly, definitions and lived experiences of sex and gender also vary across cultural and societal contexts, further complicating standardization, particularly in multinational datasets and international journals.

This interplay creates a central methodological challenge. Functional brain disorders—including mood disorders and chronic pain—are shaped by both physiological substrates and psychosocial stressors. Emerging evidence suggests that some neural networks may align more closely with biological sex, while others reflect gender‐related correlates [9]. Reporting only sex may overlook experience‐driven plasticity, whereas reporting only gender may obscure physiologically relevant mechanisms influencing disease susceptibility or treatment response.

The question, then, is not simply which variable to report, but how to do so in a way that aligns with the research question. To bridge this gap, dual reporting, listing both sex assigned at birth and self‐identified gender, has been proposed as a potential solution [10]. While conceptually appealing, this approach introduces practical challenges, including increased data complexity, sample size limitations, and uncertainty in statistical modeling and interpretation.

These challenges are especially pronounced in large, retrospective, or multicenter datasets, where definitions are often absent or inconsistent. Because researchers are often constrained by available variables, careful interpretation and transparent reporting are essential. This underscores the importance of aligning terminology with the actual data rather than retrofitting contemporary concepts onto legacy measures.

The relevance of sex and gender also varies by research context. Studies focused on neurodegenerative disease, pharmacokinetics, or structural biology may justifiably prioritize biological sex, whereas research on functional disorders, mental health, or pain syndromes may benefit from incorporating gender‐related variables that capture lived experience. However, many neuroimaging studies lie at the intersection of these domains.

It's tempting to look for a definitive rule, an editorial standard that resolves the ambiguity between sex and gender in neuroimaging. Realistically, though, such a solution seems neither to be feasible nor desirable. Where feasible, reporting both sex and gender may enhance interpretability, but this should be balanced against study design constraints and analytical feasibility. Rather than enforcing a one‐size‐fits‐all approach, the priority should be methodological clarity: clear definitions of variables, explicit descriptions of how they are measured, and justification for their inclusion.

In this context, the key challenge is not whether to use sex or gender, but how each is defined, measured, and justified. As neuroimaging moves toward more precise and individualized models of brain function, an open question remains: under what circumstances does reporting sex, gender, or both meaningfully improve interpretation and clinical relevance? The obvious follow up question is what other anatomic parts or disease models this conceptualization should be applied to in future imaging studies?

## Conflicts of Interest

M.E.S. reports consulting fees from MMI; payment for expert testimony; participation on a Data Safety Monitoring Board or Advisory Board for Ankasa, Paradigm, Spine, TOPS, Premia Spine, and Calypso; Carelon Medical board member.

## References

[1] S. Christiansen , C. Iverson , A. Flanagin , et al., AMA Manual of Style: A Guide for Authors and Editors, 11th ed. Sex/Gender 11.12.1, (Oxford University Press, 2020), accessed May 1, 2026, https://academic.oup.com/amamanualofstyle.

[2] S. Heidari , T. F. Babor , P. De Castro , S. Tort , and M. Curno , “Sex and Gender Equity in Research: Rationale for the SAGER Guidelines and Recommended Use,” Research Integrity and Peer Review 1 (2016): 2. 29451543 10.1186/s41073-016-0007-6 PMC5793986

[3] N. Alvarez‐Sanchez and S. E. Dunn , “Potential Biological Contributers to the Sex Difference in Multiple Sclerosis Progression,” Frontiers in Immunology 14 (2023): 1175874. 37122747 10.3389/fimmu.2023.1175874 PMC10140530

[4] S. Cerri , L. Mus , and F. Blandini , “Parkinson's Disease in Women and Men: What's the Difference?,” Journal of Parkinson's Disease 9 (2019): 501–515. 10.3233/JPD-191683 PMC6700650 31282427

[5] A. N. V. Ruigrok , G. Salimi‐Khorshidi , M.‐C. Lai , et al., “A Meta‐Analysis of Sex Differences in Human Brain Structure,” Neuroscience and Biobehavioral Reviews 39 (2014): 34–50. 24374381 10.1016/j.neubiorev.2013.12.004 PMC3969295

[6] D. Joel , Z. Berman , I. Tavor , et al., “Sex Beyond the Genitalia: The Human Brain Mosaic,” Proceedings of the National Academy of Sciences 112 (2015): 15468–15473. 10.1073/pnas.1509654112 PMC4687544 26621705

[7] S. E. Arambula and M. M. McCarthy , “Neuroendocrine‐Immune Crosstalk Shapes Sex‐Specific Brain Development,” Endocrinology 161 (2020): bqaa055. 32270188 10.1210/endocr/bqaa055 PMC7217281

[8] A. Guillamon , C. Junque , and E. Gómez‐Gil , “A Review of the Status of Brain Structure Research in Transsexualism,” Archives of Sexual Behavior 45 (2016): 1615–1648. 27255307 10.1007/s10508-016-0768-5 PMC4987404

[9] E. Dhamala , D. S. Bassett , B. T. Yeo , and A. J. Holmes , “Functional Brain Networks Are Associated With Both Sex and Gender in Children,” Science Advances 10 (2024): eadn4202. 38996031 10.1126/sciadv.adn4202 PMC11244548

[10] J. A. Clayton and C. Tannenbaum , “Reporting Sex, Gender, or Both in Clinical Research?,” JAMA 316 (2016): 1863. 27802482 10.1001/jama.2016.16405

